# Barriers and facilitators to knowledge translation activities within academic institutions in low- and middle-income countries

**DOI:** 10.1093/heapol/czaa188

**Published:** 2021-03-01

**Authors:** Anna Kalbarczyk, Daniela C Rodriguez, Yodi Mahendradhata, Malabika Sarker, Assefa Seme, Piyusha Majumdar, Oluwaseun O Akinyemi, Patrick Kayembe, Olakunle O Alonge

**Affiliations:** Johns Hopkins Bloomberg School of Public Health, Baltimore, MD, USA; Johns Hopkins Bloomberg School of Public Health, Baltimore, MD, USA; Faculty of Medicine, Public Health and Nursing, Universitas Gadjah Mada, Bulaksumur Yogyakarta, Indonesia; BRAC James P. Grant School of Public Health, BRAC University, Dhaka, Bangladesh; Heidelberg Global Institute of Health (HIGH), Heidelberg University, Heidelberg, Germany; Addis Ababa University School of Public Health, Ethiopia; Indian Institute of Health Management Research, Bengaluru, India; Department of Health Policy and Management, College of Medicine, University of Ibadan, Ibadan, Nigeria; School of Public Health, University of Kinshasa, Kinshasa, Democratic Republic of the Congo; Johns Hopkins Bloomberg School of Public Health, Baltimore, MD, USA

**Keywords:** Knowledge translation, organizational readiness, capacity building, motivation

## Abstract

The barriers and facilitators of conducting knowledge translation (KT) activities are well-established but less is known about the institutional forces that drive these barriers, particularly in low resource settings. Understanding organizational readiness has been used to assess and address such barriers but the employment of readiness assessments has largely been done in high-income countries. We conducted a qualitative study to describe the institutional needs and barriers in KT specific to academic institutions in low- and middle-income countries. We conducted a review of the grey and published literature to identify country health priorities and established barriers and facilitators for KT. Key-informant interviews (KII) were conducted to elicit perceptions of institutional readiness to conduct KT, including experiences with KT, and views on motivation and capacity building. Participants included representatives from academic institutions and Ministries of Health in six countries (Bangladesh, Democratic Republic of the Congo, Ethiopia, India, Indonesia, Nigeria). We conducted 18 KIIs, 11 with members of academic institutions and 7 with policymakers. KIIs were analysed using a deductive and inductive coding approach. Our findings support many well-documented barriers including lack of time, skills and institutional support to conduct KT. Three additional institutional drivers emerged around soft skills and the complexity of the policy process, alignment of incentives and institutional missions, and the role of networks. Participants reflected on often-lacking soft-skills needed by researchers to engage policy makers. Continuous engagement was viewed as a challenge given competing demands for time (both researchers and policy makers) and lack of institutional incentives to conduct KT. Strong networks, both within the institution and between institutions, were described as important for conducting KT but difficult to establish and maintain. Attention to the cross-cutting themes representing barriers and facilitators for both individuals and institutions can inform the development of capacity building strategies that meet readiness needs.

**KEY MESSAGES**

- Academic institutions in low- and middle-income countries (LMICs) face unique challenges in conducting knowledge translation (KT) activities with policy makers in their contexts. Readiness assessments can be used to describe and develop strategies to address these barriers but need to be tailored to LMICs.
- Our findings support many well-documented barriers for academic institutions in conducting KT, established largely in high-income countries. These include lack of knowledge about what KT is and how to do it; limited institutional resources (e.g. financing and time) and support (e.g. staffing and training); and the need for leadership engagement and buy-in.
- Three new themes emerged from the data as relevant for readiness for academic institutions to conduct KT in LMICs: The complexity of the policy process and need for soft-skills; A misalignment between institutional missions and incentives for researchers and policy-makers; and the nature of external and internal networks.
- The factors influencing the capacity and motivation of LMIC institutions to conduct KT activities can be used to inform the assessment of individual and institutional readiness to conduct KT in LMICs and subsequently develop capacity building strategies to address interdependent barriers and bolster facilitators.

## Introduction

A large gap continues to exist between what we know through public health research and what we do in policy and practice. However, there is increasing demand to close this ‘know-do’ gap by generating research that directly informs public health policy and practice. The field of Implementation Science emerged as a response to this need, providing guidance for researchers and implementers to consider the roles of setting, context, systems and stakeholders in adapting, packaging and facilitating the uptake of evidence to produce large-scale health impact (Alonge *et al.*, 2019).

One set of approaches used in implementation science to address the know-do gap is knowledge translation (KT) defined as ‘a dynamic and iterative process that includes the synthesis, dissemination, exchange, and ethically sound application of knowledge to improve health, provide more effective health services, and strengthen the health care system’ (Straus *et al.*, 2009). These processes, when applied rigorously and ethically, can lead to research uptake among policy makers and health practitioners.

Academic institutions are strategically placed to conduct KT activities in most settings. However, studies show that they have variable success rates in achieving this mission (Murunga *et al.*, 2020). Research conducted to understand the needs and barriers for academic institutions to conduct KT reveals a lack of knowledge of KT (what it is) (Ayah *et al.*, 2014; Harvey *et al.*, 2015; Jones *et al.*, 2015), challenges in planning and implementing KT (how to do it) (Le Gris *et al.*, 2000), weak relationships between institutions, government (Ayah *et al.*, 2014), and other stakeholders, and difficulties in communicating research effectively (Norman and Huerta, 2006). A literature review conducted by Jones *et al.* (2015) further reviewed the skills, strategies and knowledge required by researchers to conduct KT. Four themes emerged as important for improving KT efforts among researchers: (1) increased understanding of KT theory, (2) planning and implementing KT efforts in research projects, (3) developing relationships with end users throughout the research cycle and (4) communicating research effectively and engaging in two-way communication.

These identified needs and barriers in conducting KT are derived primarily from research on researchers and academic institutions in high-income countries (HICs) (Le Gris *et al.*, 2000; Norman and Huerta, 2006; Jansson *et al.*, 2010). A recent systematic review conducted by Murunga *et al.* (2020) on KT capacity and support in low- and middle-income countries (LMICs) confirmed many of these barriers for LMIC settings and indicated a need for further research into influencing factors for researchers to conduct KT at both the individual and institutional levels (Murunga *et al.*, 2020).

One approach to further understanding the institutional drivers of these barriers and developing implementation strategies is through an assessment of readiness to conduct KT (Weiner, 2009). Readiness can be both a psychological state (e.g. individual or group motivation) and a matter of capacity, i.e. skills and ability to perform a function. Institutions, like individuals, require motivation to implement interventions or widespread change (Ruhe *et al.*, 2005; Chilenski *et al.*, 2007) and in order to affect that change, an institution must also be able to complete its objectives. A recent review argues that organizational readiness in global public health is a combination of motivation and capacity which can exist at the individual, institutional and community (or environmental) levels (Dearing, 2018).

**Figure 1:**
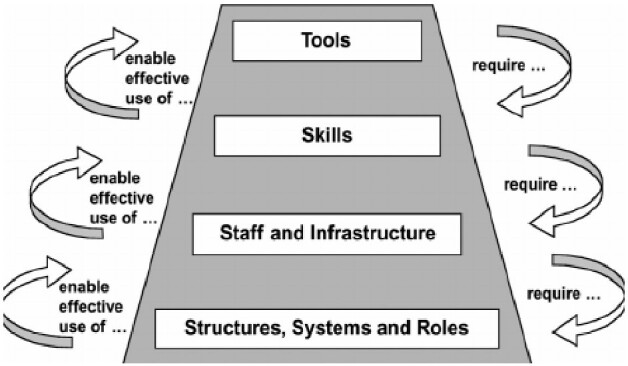
Capacity building pyramid.

Weiner argues that organizational readiness is a multi-level, multi-faceted construct influenced by internal and external contexts, culture, climate, resources, perceptions, efficacy and institutional members’ shared value for change (Weiner, 2009). This theory highlights that implementing organizational change requires collective action and recognizes the role of individual, institutional and external or societal context. Similar to the capacity building model set forth by Potter and Brough, each of these factors are hierarchical and interrelated (Figure 1) (Potter and Brough, 2004). That is, robust organizational systems and structures (e.g. culture and climate) enable the use of tools and skills that can be utilized to affect change. However, most assessments of institutional readiness to conduct KT activities have focussed on factors that capture capacity while none address motivation (Dearing, 2018); most assessments are also conducted in HICs (Weiner *et al.*, 2008).

Some of these factors and theories for assessing institutional readiness to conduct KT have been previously mapped to implementation science frameworks including Promoting Action on Research in Health Services (Gagnon *et al.*, 2014) and the Consolidated Framework for Implementation Research (CFIR) (Clinton-McHarg *et al.*, 2016; Allen *et al.*, 2017; Fernandez *et al.*, 2018; Serhal *et al.*, 2018; Walker *et al.*, 2019; Miake-Lye *et al.*, 2020). These constructs and mapping exercises are underdeveloped for LMICs and there are limited empirical studies to show which constructs are most relevant for LMICs and should therefore be prioritized.

The research presented here is part of a study designed to improve readiness for academic institutions in LMICs to conduct KT. This paper describes a qualitative study conducted to describe needs and barriers in KT specific to academic institutions in LMICs, and identify factors that are relevant for assessing institutional readiness to conduct KT activities, which can serve as the basis for developing capacity-building strategies targeted to individuals and institutions in low-resource settings.

## Materials and methods

This research is embedded in a parent project, ‘Synthesis and Translation of Research and Innovations in Polio Eradication’ (STRIPE), which seeks to map, synthesize and translate knowledge from global polio eradication efforts (Alonge *et al.*, 2020). The project is a collaboration between academic and research institutions in the USA and in seven LMICs. The six *academic* partners from the consortium (based in Bangladesh, Democratic Republic of the Congo (DRC), Ethiopia, Nigeria, India, and Indonesia) were engaged in this research.

This study included document reviews followed by key-informant interviews (KIIs). During the document reviews, institutional information was collected about the six academic partners including their institutional organograms, websites and strategic plans where available. Public information from each country’s Ministry of Health (MOH) including websites and strategic plans were also collected. Content analysis (Bowen, 2009) was performed on collected documents to understand and describe: (1) current research priorities, (2) future directions of the institutions, (3) current health status and health priorities of the country and health ministries and (4) key individuals who make decisions about research priorities at the academic institution and about the health priorities at the MOH. Data from this content analysis informed the study population and health reference points (i.e. specific health priority health issues for each country and academic institution which were used to ground the study tools).

To identify needs and barriers to KT in LMICs we also conducted a search of the peer-reviewed literature using standard search tools such as PubMed and Google Scholar. Search terms included ‘organizational readiness’, ‘knowledge translation’, ‘public health’, ‘barriers’, ‘facilitators’, ‘LMIC’ and their variants. All identified tools, reports and peer-reviewed articles were reviewed for reported barriers, facilitators and strategies for conducting KT and assessing readiness. Relevant citations contained in those articles were also reviewed.

The KIIs were designed to better understand what influences institutional readiness to conduct KT. Participants were asked about their experience with KT at their current institution including factors that could serve as barriers and/or facilitators to conducting KT activities. These activities included collecting, developing and synthesizing knowledge; utilizing knowledge-sharing platforms; disseminating knowledge to communities and decision-makers; utilizing knowledge for decision-making; and strategic communications and networking. Participants were then asked to reflect on factors that influence motivation and capacity to conduct these activities at both individual and institutional levels. Finally, participants were asked to describe capacity building strategies that had been developed, used and implemented (successfully or not) to improve KT. Suggestions for future capacity building activities and the resources needed to conduct them were also explored.

The study population included representatives from each of the six academic STRIPE institutions as well as their external government partners. Participants were identified using data from the literature review and in consultation with the six STRIPE principal investigators (PIs). Study participants from STRIPE institutions included faculty, staff or administrators who met at least one of the three following criteria:

1. Individuals involved in institutionally prioritized KT activities (health-issue specific).
2. Individuals involved in making strategic/policy decisions around KT activities at the institutional level.
3. Individuals in leadership that determine the internal context and external relationships of the institutions.

Internal participants were recruited by the research team until all three criteria were met; in many cases one individual met multiple internal criteria. External participants included policy makers engaged currently or in the past 2 years with the academic institution in efforts to conduct KT to address health issues that the institutions’ prioritized. External participants were identified through the document review and recommendations from internal participants.

KIIs were conducted in English in-person when possible and remotely via Zoom©, Skype© and Whatsapp© between November 2019 and January 2020. Interviews lasted ∼40 − 60 min. Each interview was audio-recorded, transcribed and line-by-line coded in Dedoose©, a qualitative data analysis software. Detailed notes were taken during the interview and compared with the transcript to ensure reliability of the transcription.

All data were analysed using both a deductive and inductive approach to inform the development of a quantitative tool to assess institutional readiness to conduct KT. Deductive codes were derived from two guiding frameworks including Potter and Brough’s capacity building pyramid (Potter and Brough, 2004) and the CFIR (Damschroder *et al.*, 2009). Inductive codes emerged from the data and filled in gaps where deductive codes did not capture key findings relevant to the study’s aims (e.g. ‘ownership’, ‘values’, ‘prioritization’ and ‘trust’). Comprehensive notes were utilized to ensure trustworthiness of the coding interpretations and to provide additional contextual information.

## Results

We reviewed 43 documents including recent peer-review articles published by those institutions. Strategic reports and National Health Plans highlighted country health priorities including Reproductive, Maternal and Child Health (Bangladesh, Ethiopia, Nigeria and Indonesia); Immunization coverage and family planning (DRC); Human resources for health and Health financing (India), and Universal Health Coverage (Indonesia). These national priorities were cross-checked with University strategic plans, missions, visions and core activities. Priorities that aligned between government and academic institution were considered that country’s health referent. Findings from the literature content analysis for each country/institution are presented in Table 1. Decision-makers within the MOH and the STRIPE institution related to this health priority were identified as potential participants for the KIIs.

**Table 1:** Priority health issues by STRIPE institution

Priority health issues by institution and country		
Institution and country	Prioritized health issue (referent point)	Key sources
James P Grant Brac School of Public Health, Bangladesh	Gender, sexual and reproductive health	BRAC Centres of Excellence Projects (2019); BRAC Publications (2019); National Health Research Strategy, 2009 (DGHS, 2009); Health Bulletin, 2018 (DGHS, 2018)
Kinshasa School of Public Health, Democratic Republic of the Congo (DRC)	Childhood vaccination	KSPH research projects (KSPH 2019a); KSPH recent publications (KSPH 2019b); DRC MOH Resources and Publications (2019); WHO Case Study (Ntembwa and Lerberghe, 2015)
Addis Ababa University, College of Health Sciences, Ethiopia	Reproductive, maternal, and child health	MOH Ethiopia website (2019); Setting health sector priorities: a brief overview of Ethiopia’s experience (Habtemariam and Semegn, 2018); College of Health Sciences Research Priorities, 2018 (MEPI, 2018)
IIHMR, Jaipur, India	Human resources for health and health financing	IIHMR Areas of Research (IIHMR, 2019a); IIHMR Publications, 2018, 2019 (IIHMR, 2019b); National Health Plan (2017)
Gadjah Mada University Faculty of Medicine, Public Health, and Nursing, Indonesia	Universal health coverage	WHO Country Cooperation Strategy 2014 − 2019 (World Health Organization. Regional Office for South-East Asia, 2016); Strategic Planning Ministry of Health (2015–2019)
College of Medicine, University of Ibadan, Nigeria	Reproductive, maternal and child health	National Strategic Health Development Plan (NSHDP) (TWG-NSHDP, 2009–2015); NSHDP (2018–2022)

Review of the published literature revealed a variety of documented barriers, facilitators, and strategies for institutions to conduct KT and the outcomes of KT, many of which are highlighted in the Background section of this paper.

A total of 18 KIIs were conducted across 6 countries; 5 were conducted in person and the remainder were conducted remotely. At least one internal and one external interview was held in each setting (10 internal and 8 external participants). Thirteen participants were men and 5 were women. All external participants were men except for one. Participant characteristics are shown in Table 2.

**Table 2:** Respondent’s characteristics

Country	Gender		Institutional affiliation		Total
	Male	Female	Internal	External	
Bangladesh	1	2	2	1	3
The DRC	3	0	2	1	3
Ethiopia	3	0	1	2	3
India	2	0	1	1	2
Indonesia	3	0	2	1	3
Nigeria	1	3	2	2	4
Total	13	5	10	8	18

Informants identified many factors (mainly barriers) at both the individual and institutional levels that influence KT activities. These included lack of knowledge about KT and its processes, and lack of skills to conduct KT activities; challenges procuring internal and external resources to conduct KT such as funding and software tools used for data sharing; insufficient staffing and internal structures to support KT; and lack of recognition (e.g. incentives or rewards) for successfully conducting KT. Table 3 maps these KT barriers, including illustrative quotations, to relevant CFIR constructs.

**Table 3:** CFIR level/construct, key findings and illustrative quotations

CFIR level and construct	Key findings	Illustrative quotation
Individual factors		
Knowledge and Beliefs	– Many lack knowledge of what KT is and how to do it. – Belief in the value of KT is an important motivator.	*So we have a lot of colleagues who work on [KT]. And we've been doing things perhaps not systematically. So a lot of people were not trained on KT even myself, I don't formally get training on KT* (Indonesia Internal 1). *So that's also mindset, [the faculty] don't consider that [knowledge translation] is their job* (Bangladesh Internal 1). *People have to realize that research is not just for research sake, it's not for people to just become professors and be making a lot of noise around that I'm a professor, but research, as well, for development of the society and for development in general* (Nigeria Internal 1).
Self-efficacy	– Some researchers and ministry members believe they do not have the capacity to conduct KT.	*Another one which I find in this country is what we can call a weakness in implementation capacity meaning that you can design a good intervention very clearly, but some people do not have either the mindset or the motivation to really implement the things as they were designed* (DRC Internal 2).
Inner setting		
Structural characteristics	– Lack of departmental support, namely space, training and staffing, to support KT.	*I mean, the institution should have well dedicated offices for those individuals involved in research activities, who want to sit down, analyze data, write reports, and so on. There should be well working internet facilities, well working computer facilities, and an updated software for the analysis* (Ethiopia Internal 1).
Networks and communications	– Limited connectivity between people working on similar issues or between communications staff and researchers can negatively influence capacity to conduct KT.	*It's a systemic failure. The information flow is problematic. Many people still use hard copies. You get a memo. It sits on your desk. I've been away from work for three weeks, so I don't know what I'm going to see when I resume on Wednesday, you know?* (Nigeria Internal 2). *I think getting communicators that have some knowledge of that field, as well. And then engaging the communicator right from the beginning of the process. Even with the proposal writing, ‘We are planning to do this. How do you think we should disseminate?’ Because we faculty, we always have great ideas of what we can do with the power houses and everybody else just supports us, we think. But, these people are very effective at what they do* (Nigeria Internal 1). *Well, most of the time, it's basically depends on the knowledge that an individual has on specific areas, specific area topics. But we cannot also deny the fact that connections could also work. When in some of the research you involve young people who has no experience, thinking that if you involve this young person, motivate him or her, then he or she is going to be a good researcher, a good producer, a good knowledge translator in the future. So that assumption is also be there* (Ethiopia Internal 1).
Culture (norms, values, assumptions)	– Institutional values and how they manifest through strategies and incentives are important to internal and external KT stakeholders	*I think it's having the consciousness of conducting research that's relevant to societal needs…I think in the strategic plan [of my institution], one of the objectives under research management was to conduct research at societal relevance. So, definitely that has come into the thinking of the management* (Nigeria Internal 1).
Climate	– Competing priorities and lack of institutional incentives and rewards make it difficult for people to conduct KT.	*The other thing is incentivizing individuals who are interested in conducting research and KT. The ministry should be very supportive of those individuals. But practically, I don't see such kinds of experience in the ministry* (Ethiopia, External 2).
Readiness (leadership engagement, resources, access to knowledge/info)	– Leadership engagement is needed for researchers to conduct KT but few leaders model KT activities. – Internal resources (e.g. financial, time, personnel) are rarely available to support KT. – Training not available for KT activities.	*If you don't have the buy-in of the key actors in the institution, after the research projects, the funding has gone, it's gone. So how do we make it sustainable and ensure that these activities are continued and also expanded beyond the initial focus* (Nigeria Internal 1). *Having something like a training of trainers and have people who are really into knowledge translation, really studying and doing research in that area, train people who themselves will become trainers. So some kind of meeting or workshop to train two representatives from each department, with funding to do so, you know? …at least to give them a broad overview of what knowledge translation is, and for them to see how much knowledge translation they're already doing, and how much more they want to do* (Nigeria Internal 2).
Outer setting		
Cosmopolitanism	– The degree to which an organization and its members are networked can facilitate KT.	*Of course the quality of your proposal is important. But more than that, your relationship [with the MOH] is what matters* (India Internal 1). *I think that the perception of what we do is pretty good, pretty high. Those who don't have direct contact kind of feel like professors are out of their reach, but those who do, and who meet people who are willing to break down their research into the kind of way that non-medical people and non-researchers can understand, I think they find out that there's a lot more collaboration that can take place* (Nigeria Internal 2).
Peer pressure	– Some institutions are driven to conduct KT by competition with other universities.	*And back to the issue of digital technology for telecommunications or mass communication, then this kind of role is becoming very, very competitive, okay? So if we don't do it now, then the other university will do* (Indonesia Internal 2).
External policies and incentives	– Resources (often financial) provided by funders can guide whether KT activities are done.	*Increasingly now, even donor funding looks to that because they want to show that their funding in particular has impacted on policy, or a policy* (Bangladesh Internal 2). *I think some of the decision makers, even the global politics of health, is often decided by people who haven't spent a lot of time working in developing countries. So the priorities and needs are sometimes skewed towards what the agenda is driven globally* (Bangladesh Internal 2).
Intervention characteristics		
Source	– Whether knowledge and KT processes are internal (to the country) or externally developed/funded can influence success of KT activities.	*Sometimes it depends also on where the issue comes from, if we try to disseminate certain research result, and sometimes they also look into, where is this come from? Because in some parts of the ministry…there are people who are not so keen …if they see science of interventions from international agencies* (Indonesia Internal 1).
Cost	– KT requires staffing and conducting KT can result in opportunity costs (e.g. spending time on KT vs new research or writing grants).	*[Funding organizations] don't provide any budget line for that purpose. So the institution has to invest money in their faculty to balance* (India Internal 1). *And then also providing funds, whether they be the ones to put a team together to write for grants that would aid in supporting knowledge translation, or they would redirect existing funds into that, and also make funds available for researchers to be able to tap into a pool or a pot of money that's dedicated for knowledge translation. I think that would go a long way* (Nigeria Internal 2). *The other is lack of dedicated staff, that are again, assigned for this, generally the human resource challenge. And again, even when projects are designed and agreed upon, that part of knowledge translation is overlooked, and there isn't as such, a dedicated budget for these activities* (Ethiopia Internal 1).
Process		
Planning	– Researchers and policy makers lack time to conduct KT.	*If I'm bringing in grants for the institution, if I'm bringing in money for the institution, I should probably have fewer students to supervise than my colleague who's not bringing in any grants. If I'm bringing in grants for the institution, there should be a way where that is weighted against my workload so I actually have the time to manage the grants and conduct [knowledge translation]* (Nigeria Internal 2).
Engaging	– Stakeholder engagement is key to successful KT but it can be challenging to identify, engage, and collaborate with relevant stakeholders.	*One thing is in the development case, most of the time the universities and the program experts are not sitting and discussing what are exactly the challenges on implementing the program and the like. So stakeholder involvement is one of the challenges. Stakeholders should come together to discuss the knowledge gaps, and need to find the solution* (Ethiopia External 2). *By always involving those interested, those people who are in charge of doing things, always work with them, not work for them work with them. That paradigm change must be introduced* (DRC Internal 1). *I feel the second barriers is how we can make good policy advocacy to the right people. So the strategy of delivering the content. It's not only the translating, the policy, we can do this. You've seen many mechanisms, but for delivering the message to the policy maker is at the moment, I think it more an art than a science. It's so contextually influenced* (Indonesia Internal 2)
Executing	– When KT is done, the quality, depth, timeliness and degree of engagement are viewed as important but also difficult to achieve.	*The officers that used to provide the data were also family planning providers and so sometimes they are very caught up in their work. And so submission of data timely was an issue. Timeliness was an issue* (Nigeria External 2).

Three additional themes describing institutional drivers for conducting KT that cut across the CFIR domains emerged from the data: (1) soft-skills and the policy making process, (2) misalignment between institutional missions and incentives and (3) the role of internal and external networks.

### The policy process, soft-skills, and engagement

The policymaking process, from agenda setting to policy implementation, is intricately linked to KT, whereby knowledge is developed and synthesized for policymakers to make research-informed decisions. Participants described these processes as complex while also arguing that many junior researchers naively view KT activities as straightforward. Some informants saw this disconnect between the complexities of the two processes as a potential demotivator for conducting KT and as an area for future training. Two participants from Asia described the complexity of the process and the frustrations of young scientists:

*The problem, as far as I observed, is [junior researchers] see knowledge translation more of a technocratic exercise, and in that if you have the best evidence in the world, then the policymaker would listen to you and it's very simplistic thinking. Policymaking is a political process. It's not a technical exercise where you finish your research, you transfer it and they will listen to you, it doesn't happen like that. And usually what happens is that [junior researchers] become frustrated when they try it and it doesn't happen* (Indonesia Internal 1).

*… you can't put policy in a box, and you can't put who makes policy, and how policy is made in a box. And I think we also need to unpack how we measure policies as they're made* (Bangladesh Internal 2).

Individual and institutional relationships with policy makers were viewed as integral to the success of KT because they provide channels for connecting KT and policymaking processes. Relationships could be formal or informal, depending on how the connection was made (i.e. peers at school, alumni of the institution or through past projects). One participant reflected that in their context, relationship development and maintenance is a balance:

*From an institutional perspective, the challenge is very much influenced by trust between policymakers and our institution. And that becomes tricky because that means you need a good relationship. If you get too close, then you somewhat compromise your independence, your objectivity, but if you get too far, they don't trust you* (Indonesia Internal 1).

The development of these relationships between academic researchers and policy makers was frequently described as a challenge given the complex structures of health ministries and their frequent staffing changes.

There were also some concerns that policymaking counterparts do not have the capacity to use the data to make change, even when it has been translated for them.

*Even when we do some work, do some translation, and discuss it with them, we see that people at the ministry don't have the capacity of taking it further, even using the data that we're making available to them. So most of the time, they don't use it. So I think that there is that lack of capacity within the ministry* (DRC Internal 1).

This participant further reflected that it is incumbent on the local universities to build that capacity within the MOH by generating interest in KT and providing technical training in understanding data and utilizing knowledge.

Current internal procedures at academic institutions for KT were frequently described as ad-hoc or based on individual projects and their funding sources. Many participants called for a more structured process for conducting KT at their institutions but recognized that this would require internal and external resources and dedicated staffing, e.g.:

*If you're doing anything [e.g. KT activities], you have to put your money where your mouth is. That's the bottom line. I mean it doesn't necessarily have to be a lot of money, but you have to put money towards it that will hire staff, and you have an office. And it shouldn't just be one person, one person can't do it. So, you need people who have the training, who are effective communicators and that can engage various members of staff and also train those staff* (Nigeria Internal 1).

Engagement with policy makers is one KT strategy that requires identification of and collaboration with key members of government to facilitate the policymaking process. Soft-skills [such as emotional intelligence (EQ), self- and social-awareness] were widely lauded as valuable to successfully conducting KT and engaging with policy makers but also noted as largely missing from what researchers know and what is taught in current training curricula. Two academic researchers reflected on this:

*I actually think what you need is basic EQ [Emotional Quotient] skills, which also we don't have in public health…in our training. We're not told that basically life is about treating people in a particular way, listening, being respectful. We do it only with very important people and we forget that someone in that room who is mid-level, might actually have more sway or more say* (Bangladesh Internal 2).

*I think a lot of it boils down to soft skills, being approachable. When you meet people, don't act like you're better than them in any way. Be willing to talk to people at their level. If, for instance, you meet somebody who is not in academia, and you start a conversation with them, and you find that they're struggling with English, just naturally shifting to the local language if you understand… Now they feel, ‘Oh, you're just like me’* (Nigeria, Internal 2).

Some participants argued that the best way for trainees to learn how to build these skills in KT is through active engagement and applied learning. Immersion with different stakeholders leads to an understanding of the complex policy processes and also facilitates network development.

*…learning by doing, so you send somebody to an institution where people are doing something and …work for the people there, for one month or two weeks, three weeks…In French we say ‘immersion’…Just sending somebody to a team that is doing something already and then they can learn more effectively and very quickly* (DRC, Internal 1).

### Institutional mission and incentives

When academic participants were asked if their institutions valued or prioritized KT, many had mixed responses, and reflected that the value placed on KT was changing. One researcher from Indonesia described stages of change for their institution in response to increasing demand from policy makers and funders:

*In previous times, it was more of the time of building capacity for research and good quality research. And then it moved to the stage where people are pushing more for at least disseminating in terms of scientific publication, scientific journals. And we are still at that stage but now more and more people are also questioning what are the impacts of research that we're doing? So more and more people are pushing for knowledge translation* (Indonesia Internal 1).

In contrast, external respondents, including MOH officials, tended to say ‘yes’, and that this prioritization was improving over time.

Participants also noted that prioritization of KT among academics and politicians could vary by topic, particularly when a topic is more politically charged, and the KT activities for this topic may become more prioritized (or less prioritized and scrutinized) by policymakers and/or academics and there may be misalignment of priority between policymakers and researchers.

*There are some health issues which are more politically sensitive and there are health issue which are less politically sensitive. For things which are more technical, it's usually easier for us. But when it gets to, for example, this universal health coverage then it becomes more complex because it's much more political. So different issues also will carry different challenges* (Indonesia, Internal 1).

Some leaders and senior administrators of academic institutions wavered about their institutions’ prioritization of KT, indicating that KT was included in their institutions’ missions or strategic plans but that the metrics for success were unclear. This included a lack of both institutional and individual metrics, and inconsistencies in how these metrics are applied where they exist. Multiple participants across different contexts described this misalignment, particularly in relationship to promotion pathways within their institutions:

*No, [knowledge translation]is often the last thing and it's not clearly defined. I think I have been at the University for over 10 years. If I could understand what exactly is community service? And then now they are saying, ‘Well we're including [community service] in the promotion guidelines and we've added a score’. But, if you don't produce articles, if you don't publish, no matter how much community service you may do, if you don't get the scores for publications, you're not going anywhere* (Nigeria Internal 1).

*You have to understand that translating knowledge per se is not one of the priorities of people… I can do research, publish it, so I can get a promotion. It's not really translating it, that knowledge, for the improvement of health within the country* (DRC Internal 1).

*And the main barrier is that most of our faculty members, by rule by regulations, they have to do teaching, service, and research, meaning they only get limited time to do research and actually not have any time for knowledge translation. It doesn't count basically…you don't get credit* (Indonesia Internal 1).

While promotion pathways for KT remained unclear, academic participants offered many suggestions for potential alternative incentives to motivate members of their institution to conduct KT. This included financial incentives (e.g. bonuses); career development opportunities such as attending international conferences and trainings; providing supplies, infrastructure, equipment and logistics needed to conduct KT activities; and developing robust online tools and platforms for data sharing and translation.

Participants also noted that while KT was included in their institutional missions, these activities faced competing priorities from other institutional missions including the generation of knowledge through research and knowledge dissemination through student learning. For example,

*The university has a triple mission. First of all, to train then to conduct research. And then the third is to serve the community. So far what we have seen is that the university is doing little I would say, on that third mission, it's not very well developed. That's where the translation of research can really play a role* (DRC Internal 2).

Given these competing priorities, time emerged as a consistent limiting factor for conducting KT activities both among internal and external participants. One respondent discussed how KT activities, when done well, are welcomed by the MOH as a time saver:

*Ministry people are very busy, but they have to get things done. They don't have time for all the processes of analysis and derivatives, but anybody who has solved their problem, provided analysis, and feedback, they're welcome [by the ministry]* (India Internal 1).

MOH respondents also reflected on the limited availability of researchers. However, despite recognizing these barriers, multiple external stakeholders declared that the ultimate goal of academic institutions should be to support the ministry of health and that this should be the primary priority of any public academic institution.

*The final goal of [the academic] institution should be especially supporting the ministry and the program. And at the end they should analyze and advise based on the gap they have* (Ethiopia External 1).

Some participants also described the impact of KT activities on health programs, policies, and outcomes as important incentives for conducting KT work for themselves, junior researchers, and ministry members:

*People can see it and people can feel the change in themselves when they’re really able to help people solve their own problems. If you can change their health, change practices, then it is a win-win situation, both for the people conducting knowledge translation and for the community served* (India, Internal 2).

*[MOH officials] look forward to hearing more about the indicators because [our program] provided health related indicators and they always wanted to know how their state was doing… I think our activities were very well-received, and I think that they used the data as best as they could. I do know that they used it for their cost and implementation plan, and I heard that in many of their meetings, they quote our data* (Nigeria, Internal 2).

### Depth and breadth of networks

Participants viewed the strength and nature of networks as an influencer on both institutional and individual capacity to conduct KT. These included peer-to-peer interorganizational networks and external networks with other institutions such as the MOH, funders, non-profit organizations or other academic institutions. Strong internal networks provide access to robust teams and much needed human resources in addition to access to key external decision-makers (e.g. senior members of the MOH).

Strong external networks facilitate acceptance of KT, co-operation and trust, and provide access to financial resources needed for KT.

*Because we are a long standing university, very respected, with long-term relationships [with the MOH], the acceptability by the government, by stakeholders, donors is much, much higher than with the other new universities…Whether you are to be involved in health, or in other sciences, or in other social sciences, the university is very much acknowledged and accepted* (Ethiopia, Internal 1).

Institutional reputation among the community and the MOH was seen as an important factor that influences cosmopolitanism and KT activities. At least one respondent from each academic institution described there being value in the institution’s age or status within the country. Participants similarly noted that many alumni served in the MOH, strengthening the connection between the university and government.

*We are fortunate because we are the oldest university in a country and with a lot of interaction with the government alumni, the current minister is our alumni [sic]. Because these are people who actually have some relations with our faculty members when they were going to university together at the same class and so forth…To a certain extent, I think we're at better position than other universities in that we have a better insight into the government* (Indonesia Internal 1).

*Because BRAC has a good name, it's well established and all of our graduates are employed, many are in government. There are many different key positions globally, but also in the country. So that helps widen our networks* (Bangladesh Internal 2).

Some external participants indicated that decisions to collaborate or reach out to an academic institution for KT were based more on an institution’s capacity and expertise on the topic of interest than on well-established partnerships. One interviewee in Nigeria said:

*From the ministry perspective, you do not just engage anybody just because you're a lecturer or you're in higher academic institution. There has to be terms of reference and value for money. You must add value to the program or project…if you don't have the capability, there's no point engaging* (Nigeria External 1).

Time emerged again as important for building and sustaining networks. Consistent engagement over time, whether strategic or more organic, was viewed as valuable to strengthening networks needed for conducting KT and served to build trust between researchers and policy makers.

*Another thing would be to make sure that you are available. Sometimes people give you their cards, they send you emails, you don't reply, they try to call you, you don't pick up your phone, you don't respond to text messages. Many of those things put people off, because I've heard people complaining to me before, and I've heard people jokingly say, ‘Are you always online or something?’ Which is not true. Sometimes I'm not available myself, but I do try* (Nigeria Internal 2).

*For me it's been living and working here for a long time, understanding context. We're academics in our work, researchers, we do a lot applied work. It's building relationships. A lot of relationships are built on trust* (Bangladesh Internal 2).

Enabling institutional environments with support and knowledgeable leadership was explored as an approach to improving networks and opportunities for conducting KT, such as institutions providing appropriate tools, funding, time, and mentorship opportunities, both formal and informal.

*I think having mentors that consider knowledge translation important and that prioritize knowledge translation would be a great motivating factor. Mentors, as well as senior colleagues…but just seeing other people doing it, and seeing how they do it, and potentially how it can be rewarding is helpful and encouraging individual researchers to also conduct knowledge translation* (Nigeria Internal 2).

*Just to give you an example, just a few months ago, we have a new government. They have a new national medium-term plan for the next five years, and they asked me for inputs. And instead of just me, then I gather colleagues and just have a discussion of what are their inputs based on their studies, which can be used to be considered for the next five years national medium-term plan for health* (Indonesia Internal 1).

The role of internal networks and team development to tackle complex health issues and conduct KT activities were viewed as important but sometimes lacking. Some participants described the diversity of the teams and what is required to do KT well:

*Because [KT] is so multi-dimensional you need economist, you need a demographer, you need social scientist, you need basic scientists. They're all necessary. And then there should be a language editor and that’s not a minuscule kind job. And then the grant management to look at the budget and reporting documents which are required* (India, Internal 1).

Again, participants noted the need for more human resources and stronger internal communications and advocacy departments dedicated to supporting researchers in policymaker engagement.

Institutional leaders were an important human resource, also seen as playing a valuable role in supporting KT activities and teams. One external participant described the importance of transformational leadership in KT:

*Transformational leadership means that the teamwork is there so we not do the work individually. One leader will delegate his power to his team member. The team members will be empowered to do more things to do or similar things like leader. So, this is a kind of teamwork, good effective teamwork, where the leader transfers his quality to other team members* (Bangladesh External 1).

## Discussion

KT activities, when conducted rigorously, have been shown to have significant impact on health policies and programs in low-resource settings. This research identified many well-documented barriers to conducting KT activities (Le Gris *et al.*, 2000; Norman and Huerta, 2006; Ayah *et al.*, 2014; Harvey *et al.*, 2015; Jones *et al.*, 2015; Murunga *et al.*, 2020) that have previously been described for HICs and LMICs including a lack of knowledge about what KT is and how to do it, limited resources (e.g. time and funding) and institutional support (e.g. staffing and infrastructure) for KT, and the need for buy-in from members of leadership.

Our approach uniquely utilized the six STRIPE institutions as the level of analysis, shifting the focus from individual influencers to organizational drivers of KT to understand *why* these documented barriers and facilitators exist—i.e. what drives the positive and negative impacts they have? By virtue of their membership in the consortium, each institution is well-positioned to conduct health research, collaborate with external institutions and disseminate findings. However, the extent to which these activities are possible at the institutional level and in collaboration with the MoH, regardless of project or investigator, varies widely by context. Three additional emergent themes or drivers resonated across the CFIR and across these six LMIC contexts. These included the complexity of the KT and policy processes and need for soft-skills; the role of institutional missions and incentives; and the value and challenges in developing robust internal and external networks. The complexity of the policy process requires soft skills to navigate this process and engage with policymakers, for which academics often lack and rarely receive training. Concurrently, institutions lack the strategy needed to continuously conduct KT, even if KT is integral to their institutional missions. The beneficial role of networks, both internal and external to the institution, cannot be overstated, and serve to facilitate acceptability of knowledge generated through research and its utilization in policymaking. KT processes can also create an array of opportunities for academic institutions and researchers. Diverse stakeholder engagement may lead to new research questions that address national priorities, result in policy shifts and build relationships. These external networks are highly valuable for individuals and institutions but may be challenging to acquire. Interestingly, some academic participants noted that they sometimes rely on and leverage relationships with alumni (part of their internal networks) who serve in the MOH to increase access to policy makers (external networks). While this can be a positive networking tool, it also speaks to policy elites and actor coalitions that may control networks and fragment health governance (McDougall, 2016; Shiffman *et al.*, 2016).

Many of these themes and sub-themes are intricately linked and can have multiplicative effects on capacity and motivation, the core components of readiness. Strong networks are invaluable for KT activities but require ongoing engagement and time to maintain; across contexts, time was limited for both researchers and policy makers, particularly when existing incentives are aimed at other activities. An institutions’ internal network and extent to which teams can form and collaborate is dependent on how the institution prioritizes its resources (e.g. staffing, funding and leadership input) and how members of the leadership engage with the policy process. Capacity building efforts to improve KT in these settings must account for these linkages by addressing needs for internal and external individuals and their institutions. Further, this research underscores the potential for academic institutions to support the MOH and build their capacity to conduct KT, though that is not always within their remit.

Study participants reflected on capacity issues at multiple levels: from a lack of training and skills (among both researchers and policy makers) to conduct KT and navigate the policy environment to insufficient institutional infrastructure and staff. Using the Potter and Brough (2004) capacity building pyramid framework we can better understand the inter-relatedness of these capacity challenges. For example, researchers interested in conducting KT who have clearly defined roles and responsibilities, supportive staff, and clear mechanisms for communication and dissemination will be enabled to utilize their knowledge and skills in KT activities. Each of these capacity gaps reflect potential capacity building opportunities specific to the needs of researchers and their institutions in LMICs. To fully support KT activities in LMIC settings we need to account for these factors as capacity building strategies are developed and implemented. Some groups have proposed and evaluated capacity building strategies for improving KT and other dissemination and implementation research activities. Brownson *et al.* (2017) shared their strategies and lessons including spending time with institutional leadership to garner institutional commitment for institutional changes; promoting active participation across multidisciplinary teams; incorporating networking activities in training programs; and developing data systems for ongoing evaluation (Brownson *et al.*, 2017). A recent literature review sought to identify core KT competencies and reported on interventions to improve these competencies such as hands-on training, educational sessions, leadership and communication strategies, and funding a KT champion (Mallidou *et al.*, 2018). Mentorship models have also been explored as a strategy to build capacity for KT (Gagliardi *et al.*, 2009) and were recognized by KII participants as valuable for improving engagement, the execution of KT, and the establishment of networks (Table 3). El-Jardali and Fadlallah propose a framework for KT where capacity building for researchers is central, emphasizing that efforts should target the individual, team, institutional and systems levels (El-Jardali and Fadlallah, 2015). While institutional capacity has been acknowledged as fundamental to promoting sustainable use of evidence, there is less appetite for and evidence on how to conduct institutional capacity building activities (Hawkes *et al.*, 2016); in low-resource settings, many institutions may not be ready to conduct them (Ayah *et al.*, 2014).

Readiness has often referred to an individual psychological state of motivation and plays an important role in many theories of behaviour change including the Health Belief Model (e.g. self-efficacy) (Strecher *et al.*, 1997), Prochaska’s Stages of Change Model (e.g. determination) (Prochaska *et al.*, 2002) and Social Cognitive Theory (e.g. capability and self-efficacy) (Bandura, 1986). Assessing readiness to conduct KT activities is a key strategy that can help identify institutional drivers prior to conducting a specific KT activity in an academic institution. Our results describe many possible motivators and demotivators including lack of training and institutional support, a complex policy process that requires nuanced approaches and dedicated time, and lack of networks for junior researchers that impact readiness to conduct KT activities. At the institutional level, motivation was most often described in terms of prioritization; if an institution prioritized KT then members of that institution would also prioritize KT activities. These motivators can be used to bolster readiness tools that until now, largely focus on capacity only (Dearing, 2018). Given the dearth of comprehensive readiness tools designed for low-resource settings, data from these KIIs will be used to develop and adapt items related to capacity and motivation for a quantitative readiness assessment, responsive to KT in LMIC contexts.

This paper also furthers the theory on how to develop institutional-level interventions for KT in LMICs. Murunga *et al.* (2020) identified barriers for conducting KT to include KT knowledge and skills of target audience (target audiences lack knowledge/understanding, and skills related to research and policy development), research institutional support (inadequate institutional support and incentives for KT), and researcher/target audience collaboration and networking, among others. A deeper look at these barriers suggests that they are inter-dependent—and may be driven by the interactions of two or more cross-cutting themes identified in this paper, e.g. ‘alignment of incentives and institutional missions’ and ‘role of networks in KT’. Institutions that do not identify KT as priority will not develop networks that support KT, and are less likely to prioritize raising awareness of policymakers to demand KT. Therefore, where intervention resources are limited, it might be efficient to address these cross-cutting themes as higher-order intervention levers than to focus on individual barriers. For example, addressing institutional missions and culture (through strategies such as instituting transformational leadership at academic institutions, the formation of multidisciplinary and cross-generational working groups, and lookback assessments to reveal failures and missed opportunities of the institutions to make impact) may yield significant positive externalities that would impact multiple barriers at the same time.

This research is subject to certain limitations. While members internal and external to the academic institutions participated in each country, recruiting national policy makers working on priority areas was a challenge; in most settings only one MOH official participated, providing a limited perspective on these issues from the external point of view. These six countries also offer a snapshot of the overall experience in LMICs. All team members contributed to the data interpretation, though only one member of the team conducted and analysed the interviews. While this ensures data were collected and coded consistently it raises concerns with reflexivity, recognizing that the researchers’ background affects the angle of investigation and interpretation of results (Malterud, 2011). However, this is counteracted by the sequential exploratory design of the study and the researcher’s acknowledgement of her bias and experience.

KT activities have the potential to decrease the ‘know-do’ gap, bringing evidence-based interventions into policy and practice through rigorous, iterative approaches. For KT to be effective, individuals and institutions require both motivation and capacity for KT. Attention to the complexity of the policy process, the role of networks and the need for soft-skills to promote continuous engagement with training, mentorship, and network development strategies could help facilitate effective KT activities by academic institutions in LMICs. Each of the cross-cutting themes can be used to guide readiness assessments and subsequent development of additional capacity building tools and strategies for KT in LMICs.

## Data availability

Data available upon request.

## Funding

The STRIPE consortium and its activities are funded by the Bill and Melinda Gates Foundation.
